# Electrical and dielectric parameters in TiO_2_-NW/Ge-NW heterostructure MOS device synthesized by glancing angle deposition technique

**DOI:** 10.1038/s41598-021-99354-1

**Published:** 2021-10-06

**Authors:** H. Manas Singh, Ying Ying Lim, P. Chinnamuthu

**Affiliations:** 1 Department of Electronics and Communication Engineering, NIT Nagaland, Dimapur, 797103 India; 2 Department of Electrical, Electronic and Communication Engineering, Tokyo City University, Tokyo, 158-8557 Japan

**Keywords:** Nanowires, Nanoscale devices

## Abstract

This paper reports the catalyst-free coaxial TiO_2_/Ge-nanowire (NW) heterostructure synthesis using the glancing angle deposition (GLAD) technique integrated into an electron beam evaporator. The frequency and voltage dependence of the capacitance–voltage (C–V) and conductance–voltage (G/ω–V) characteristics of an Ag/TiO_2_-NW/Ge-NW/Si device over a wide range of frequency (10 kHz–5 MHz) and voltage (− 5 V to + 5 V) at room temperature were investigated. The study established strong dependence on the applied frequency and voltage bias. Both C–V and G/ω–V values showed wide dispersion in depletion region due to interface defect states (D_it_) and series resistance (R_s_). The C and G/ω value decreases with an increase in applied frequency. The voltage and frequency-dependent D_it_ and R_s_ were calculated from the Hill-Coleman and Nicollian–Brews methods, respectively. It is observed that the overall D_it_ and R_s_ for the device decrease with an increase in the frequency at different voltages. The dielectric properties such as dielectric constant ($\upepsilon$′), loss ($\upepsilon$″) and loss tangent (tan δ) were determined from the C–V and G/ω–V measurements. It is observed that $\upepsilon$′, $\upepsilon$″ decreases with the increase in frequency. Therefore, the proposed MOS structure provides a promising alternative approach to enhance the device capability in the opto-electronics industry.

## Introduction

One-dimensional (1D) nanostructures like nanowires and nanorods have attracted huge interest over the last few decades in the field of metal oxide semiconductor (MOS) for various applications like photodetectors^1–6^, sensors^7,8^, photovoltaic systems^9^, non-volatile memory applications^10,11^ etc. Following the Moore’s law, the current semiconductor based MOS devices are facing a technological limitation in the form of scalability and leakage current. In order to further improve the MOS device performance, the International Technology Roadmap for semiconductors (ITRS) for future technology has recommended the use of a high mobility material as an alternative solution. In this context, recent papers have reported Germanium (Ge) with high-k dielectric based MOS devices which show good overall performance^12,13^. This is because Ge has high electron and hole mobility compared to silicon (Si) and a low band gap which enables operation at low voltage^14^. This feature, together with the opportunity to co-integrate Ge with high-k dielectrics such as Al_2_O_3_, HfO_2_, TiO_2_ etc., makes Ge a practical candidate for future MOS device applications. However, the integration of Ge with a MOS capacitor is challenging due to the native unintentional formation of GeO_x_ which is unstable compared to SiO_2_. This issue is mitigated by the integration of titanium dioxide (TiO_2_) which is a high dielectric material with Ge. The unstable GeO_x_ is reduced by the diffusion of Ge into the TiO_2_. At the same time, rutile-TiO_2_ can be formed which has a high-dielectric constant^15^. Recent reports suggests that this approach can be achieved by various synthesis techniques for different applications^16–18^. Amongst these synthesis techniques, the glancing angle deposition (GLAD) is a catalyst free environment friendly technique which can synthesize numerous materials for various applications^2,19–21^. Furthermore, vertical nanowires can be easily achieved with the GLAD technique compared to other available techniques^22^.

In this work, a Ag/TiO_2_-NW/Ge-NW/Si (MOS) device is synthesized using the GLAD technique integrated into an electron-beam evaporator. The frequency and voltage dependence capacitance (C) and conductance (G/ω) at room-temperature are analyzed. The aim of the work is to investigate to the effect of interface state density (D_it_), series resistance (R_s_) on the C–V and G/ω–V characteristics as well as the dielectric parameters of the proposed MOS device with the applied bias voltage and frequency.

## Results and discussion

**Figure 1:**
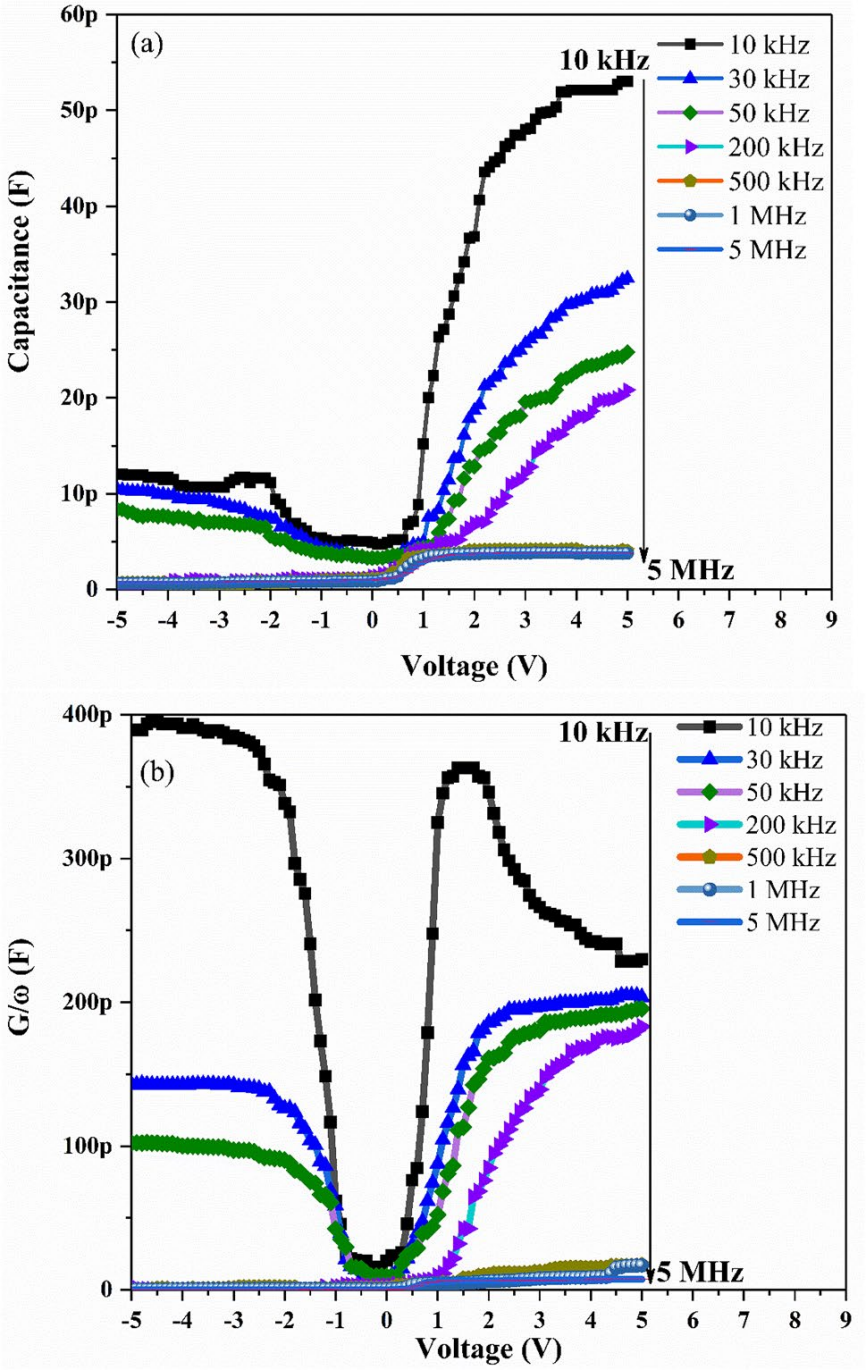
Plots of the (**a**) C–V and (**b**) G/ω–V for the Ag/TiO_2_-NW/Ge-NW/Si (MOS) device.

**Figure 2:**
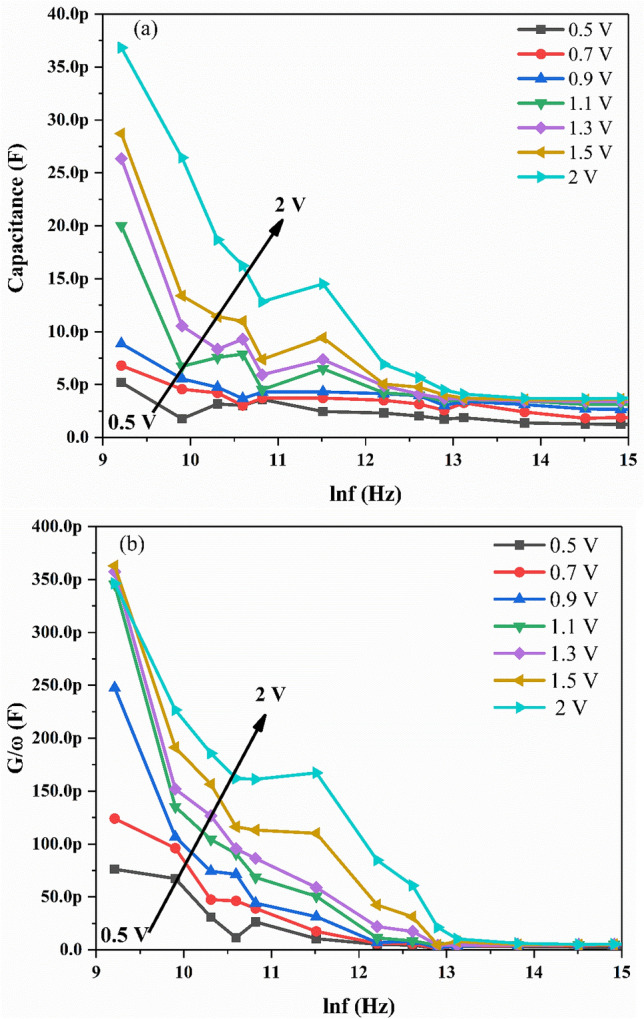
Plots of the (**a**) C-lnf and (**b**) G/ω-lnf for the Ag/TiO_2_-NW/Ge-NW/Si (MOS) device.

The C–V and G/ω–V measurements of Ag/TiO_2_-NW/Ge-NW/Si MOS device are obtained for different frequencies and voltage ranges at room-temperature as shown in Fig. 1a,b. It is observed from Fig. 1a, that the MOS device displayed extensive distribution in depletion, inversion and accumulation regions. As seen in the figure, the MOS device showed an inversion region (-5 V to 0 V), a depletion region (0 V to 2 V) and an accumulation region (2 V to 5 V) at almost each frequency respectively. The voltage shift is due to the presence of surface states in the MOS device^23^. The measured capacitance (C) and conductance (G/ω) values showed strong dependence on frequency and voltage in the depletion region which might be due to the D_it_ and R_s_ in the MOS device. The decrease in C and G/ω with the increase in frequency as shown in Fig. 2a,b, might be because the D_it_ cannot follow the alternating current (ac) signal at high frequencies and hence the contribution of these states to capacitance and conductance decrease with the increase in frequency^24^. This makes the contribution of interface state capacitance to the total capacitance negligible^25^. Thus, the measured C–V and G/ω–V values are close to the ideal case at high frequencies. At low frequency, the wide dispersion present in depletion region for both the C–V and G/ω–V curves is due to the existence of D_it_^26^.

**Figure 3:**
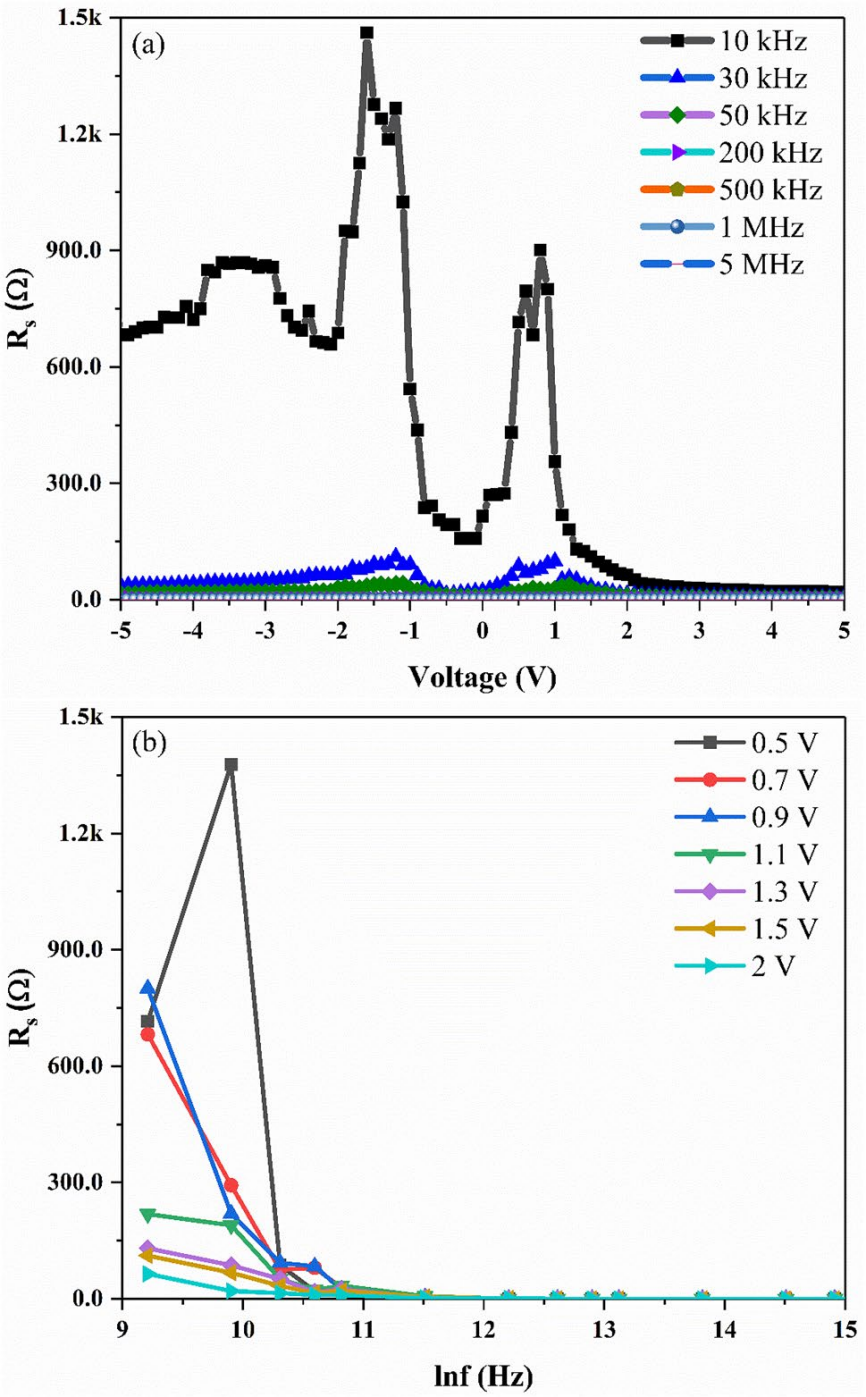
Plots of (**a**) R_s_–V and (**b**) R_s_-lnf for the Ag/TiO_2_-NW/Ge-NW/Si (MOS) device.

The electrical parameters D_it_ and R_s_ could have attributed to the deviation in both the C–V and G/ω–V curves from the ideal behaviour. The unsymmetrical growth of nanowires due to the shadowing effect inherent in the GLAD technique may have induced inhomogeneous contact during the metallization process between the metal contact and the semiconductor junction. This also could be the reason for the deviation from the ideal behaviour of the MOS device. The Nicollian and Brews method^25^ is used to determine the parameters R_s_ in the measured voltage range of the MOS device. This method is reported to be more precise in comparison with Norde and Cheung functions and conductance and admittance methods as per previous report^26^. R_s_ is calculated using the Eq. (1) ^25^ given below:

1 $$R_{s } = \frac{{G_{ma} }}{{G_{ma}^{2} + \left( {\omega C_{ma} } \right)^{2} }}$$

where C_ma_ and G_ma_ are the measured C and G for any biased voltage and ω is the angular frequency. Figure 3a,b, show the R_s_–V plots determined from Eq. 1. It is observed from Fig. 3a that R_s_ decreases with an increase in frequency and the presence of peak around − 0.5 V to 1.3 V at low frequencies is due to the presence of D_it_. The changes in R_s_ from region to region clearly shows that R_s_ is dependent on both the applied frequency and voltage. The change in R_s_ is evident especially in the inversion and depletion regions from the low to the high frequency range. It should be noted that R_s_ is independent of frequency at the accumulation region from frequency greater than 30 kHz. Furthermore, the voltage dependent R_s_ show almost constant value at higher frequencies (frequency > 200 kHz). This shows that for Ag/TiO_2_-NW/Ge-NW/Si (MOS) device, the R_s_ is effective in the accumulation region in high voltage at high frequencies (frequency > 200 kHz).

The D_it_ is another parameter which affects the measured C–V and G/ω–V for the Ag/TiO_2_-NW/Ge-NW/Si (MOS) device. The frequency dependent D_it_ plot can be obtained using the Hill-Coleman method from Eq. (2)^27^ given below:

2 $$D_{it} = \frac{2}{qA}\frac{{\left( {\frac{{G_{m} }}{\omega }} \right)_{max} }}{{\left( {\frac{{\left( {\frac{{G_{m} }}{\omega }} \right)_{max} }}{{C_{ox} }}} \right)^{2} + \left( {1 - \frac{{C_{m} }}{{C_{ox} }}} \right)^{2} }}$$

where C_ox_ and (G_m_/ω) _max_ are the interlayer capacitance, maximum value of conductance which is corresponding to C_m_ and the value of C_ox_ can be calculated from the measured C and G/ω values at the strong accumulation region using Eq. (3)^28^ as given below:

3 $$C_{ox } = C_{ma } \left( {1 + \left( {\frac{{G_{ma} }}{{\omega C_{ma} }}} \right)^{2} } \right)$$

**Figure 4:**
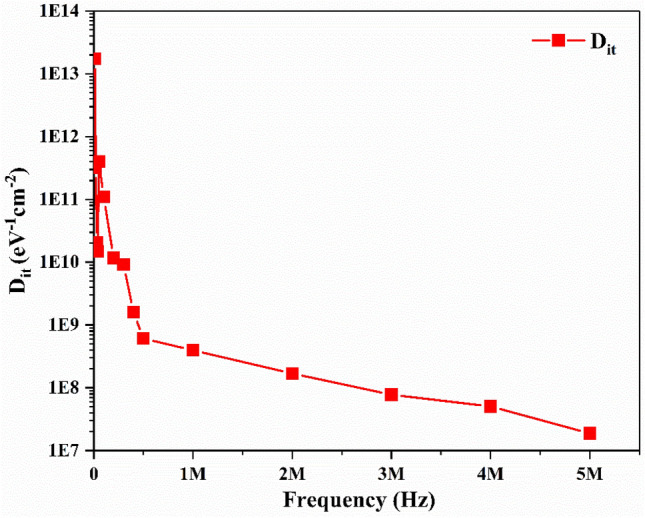
Frequency dependent D_it_ plot for the Ag/TiO_2_-NW/Ge-NW/Si (MOS) device.

The frequency dependent D_it_ determined using Eqs. 2 and 3 is shown in Fig. 4. It is observed that the D_it_ decreases with an increase in frequency for the Ag/TiO_2_-NW/Ge-NW/Si (MOS) device which is due to the low power follow rate at high frequencies^28^. Furthermore, the obtained D_it_ value at high frequency ( frequency > 400 kHz) is found to be better in comparison with the reported values^17,29,30^ and thus addresses the serious issue of high interface state densities. The distinct peak seen in the D_it_ plot in Fig. 4 (inset) at 100 kHz and 300 kHz having D_it_ values of 1.73 × 10^13^ eV^−1^ cm^−2^ and 2.05 × 10^10^ eV^−1^ cm^−2^ with defect lifetime of ~ 10 μs and 3.3 μs, respectively. The difference in the lifetime of the defects observed from the D_it_ plot is primarily due to the slow traps and fast traps also known as interface traps corresponding to large defect lifetime and small defect lifetime, respectively^29^. It can be seen that the maximum value of C_m_ decreases with an increase in R_s_ which is in good agreement with the theoretical results stated by Chattopadhyay et al.^31^. Therefore, D_it_ at low frequency in the Ag/TiO_2_-NW/Ge-NW/Si (MOS) device can follow the ac signal and yield an excess capacitance, thus resulting in D_it_ being more pronounced compared to R_s_. In contrast to the low frequency values, the D_it_ values at high frequency cannot follow the ac signal which makes the contribution of interface state capacitance to total capacitance negligible. This results in the contribution of R_s_ being more pronounced.

In order to obtain the corrected capacitance (C_c_) and corrected conductance (G_c_/ω) at different frequencies both measured C and G/ω are corrected considering the effect of series resistance (R_s_) using the Eq. (4–6)^28^ given below:

4 $$C_{c } = \frac{{\left[ {G_{m}^{2} + \left( {\omega C_{m} } \right)^{2} } \right]C_{m} }}{{a^{2} + \left( {\omega C_{m} } \right)^{2} }}$$

5 $$G_{c} = \frac{{G_{m}^{2} + \left( {\omega C_{m} } \right)^{2} a}}{{a^{2} + \left( {\omega C_{m} } \right)^{2} }}$$

6 $$a = G_{m} - \left[ {G_{m}^{2} + \left( {\omega C_{m} } \right)^{2} } \right]R_{s}$$

**Figure 5:**
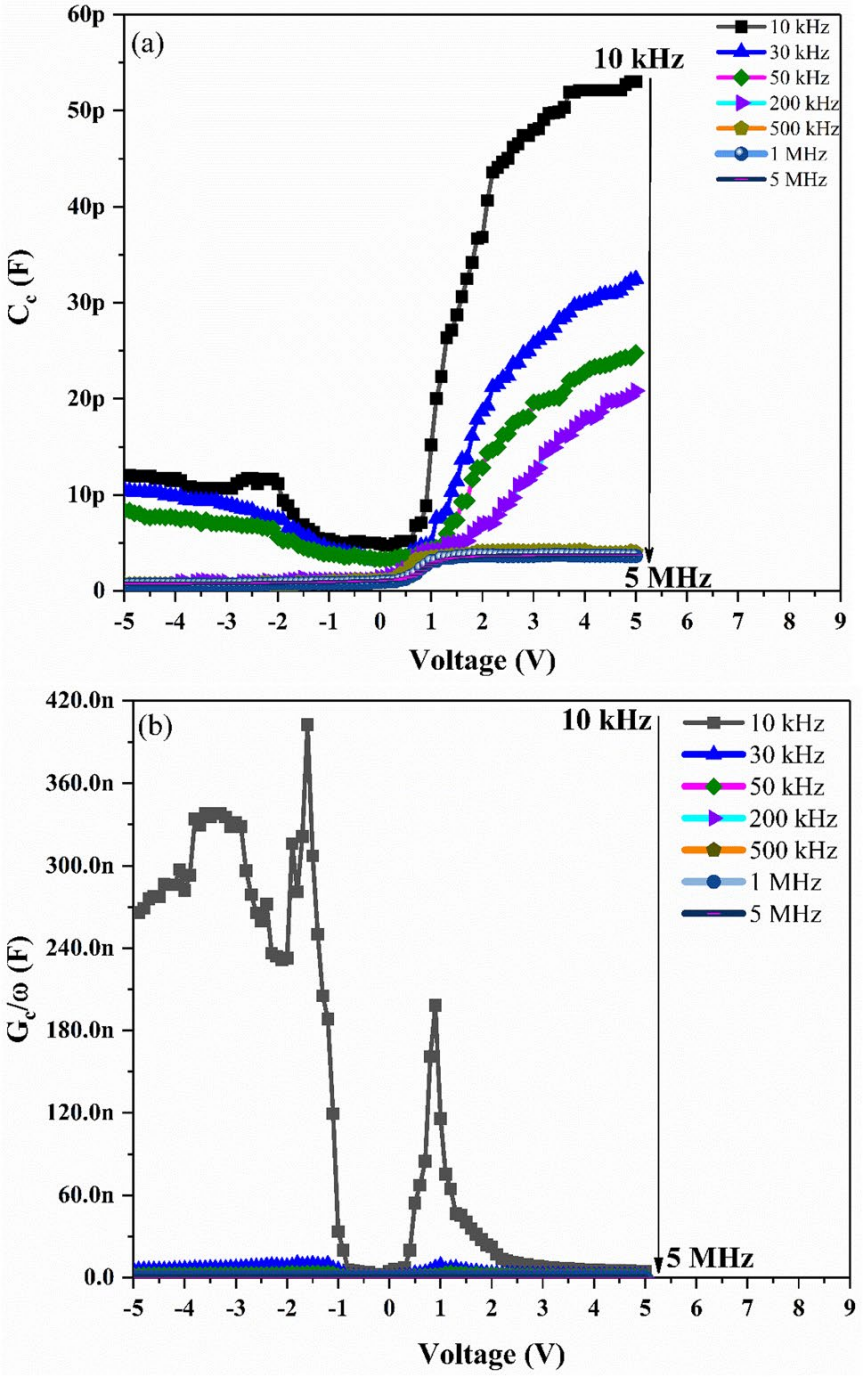
Plots of the (**a**) C_C﻿-V_ and (**b**) G_c_/ω–V for the Ag/TiO_2_-NW/Ge-NW/Si (MOS) device.

Figure 5 represents the C_C–V_ and G_c_/ω–V plots for the Ag/TiO_2_-NW/Ge-NW/Si (MOS) device. It is observed after making the correction that is considering the effect of R_s_ in C_c_ versus V plot, there is no significant change in the corrected capacitance (C_c_) values. However in the case of the corrected conductance (G_c_), it is observed that there is a decrease in the C_c_ value with an increase in frequency and the existence of peaks in the G_c_/ω–V plot confirms the charge transfer taking place at the interface^28^.

The dependence of the dielectric constant ($\upepsilon$′), dielectric loss ($\upepsilon$″) and dielectric loss tangent (tan(δ)) on the frequency and voltage are investigated in various frequencies (10 kHz to 5 MHz) at different voltage ranges (-5 V to 5 V) at room-temperature. The values of the dielectric constant ($\upepsilon$′) and dielectric loss ($\upepsilon$″) of the Ag/TiO_2_-NW/Ge-NW/Si (MOS) device are obtained using the measured C, G/ω, thickness of the oxide layer, area of diode and permittivity of free space ($\upepsilon$_o_). The complex dielectric constant ($\varepsilon * = \varepsilon ^{\prime } - j\varepsilon ^{{\prime \prime }}$), the real and imaginary parts could then be determined from the relations given in Eq. (7–8)^32^ given below:

7 $$\varepsilon ^{\prime } = \frac{{C_{{ox}} }}{{C_{o} }}$$

8 $$\varepsilon ^{{\prime \prime }} = \frac{{d_{{ox}} }}{{A\varepsilon _{o} }}\frac{{G_{m} }}{\omega } = \frac{{G_{m} }}{{C_{o} \omega }}$$

where $C_{o} = \varepsilon _{o} \left( {\frac{A}{{d_{{ox}} }}} \right)$; A is the area of the device, d_ox_ is the oxide layer thickness, $\upepsilon$_o_ is the permittivity of free space ($\upepsilon$_o_ = 8.85 × 10^–14^ F/cm), G_m_ is the conductivity of MOS structure and ω is the angular frequency and j is the imaginary root of − 1. The dielectric loss tangent can be determined by the relation given in Eq. (9)^32^ given below:

9 $$\tan \delta = \frac{{\varepsilon ^{{\prime \prime }} }}{{\varepsilon ^{\prime } }}$$

**Figure 6:**
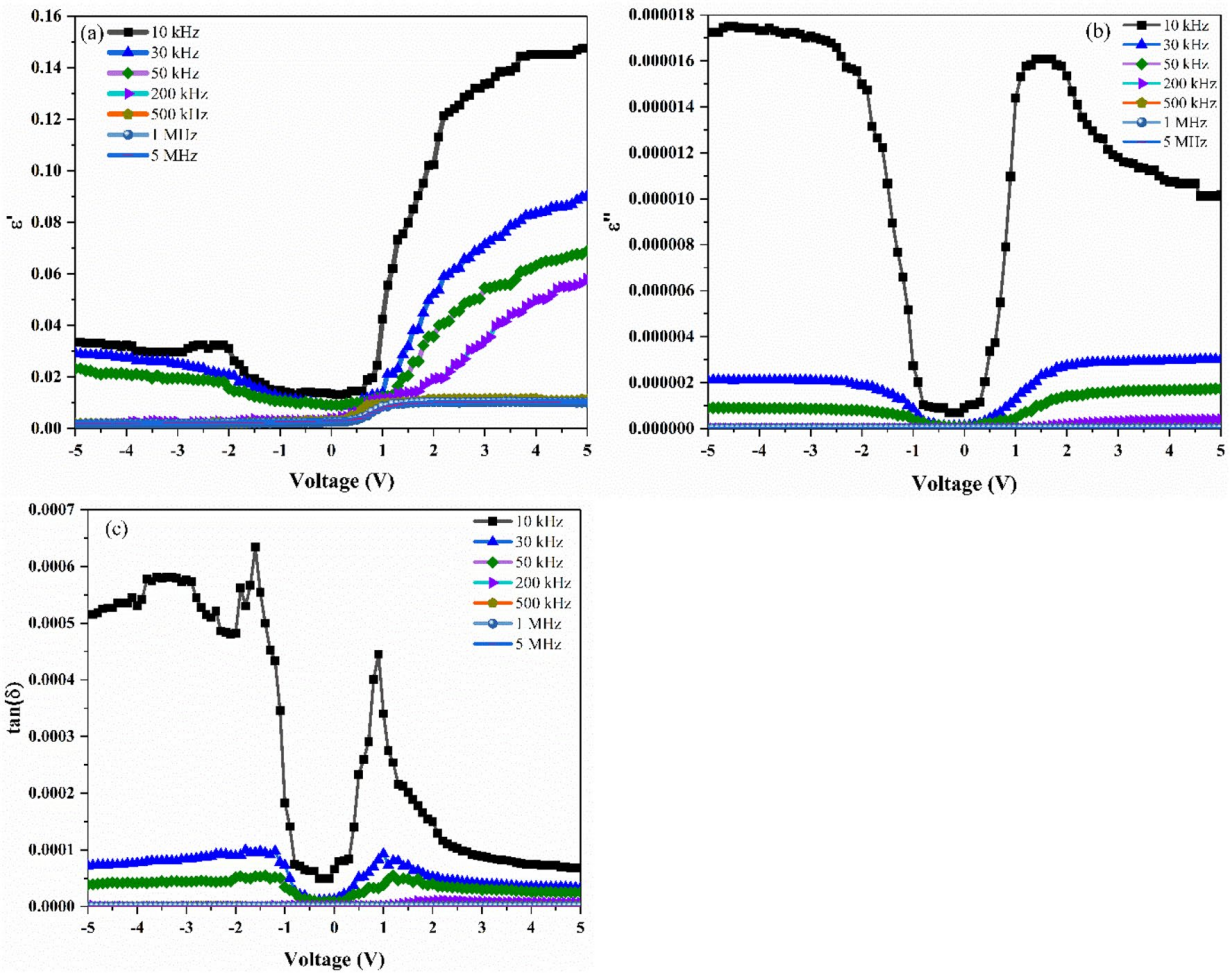
Plot of (**a**) $\upepsilon$′–V, (**b**) $\upepsilon$″–V, (**c**) tan(δ)–V for the Ag/TiO_2_-NW/Ge-NW/Si (MOS) device.

**Figure 7:**
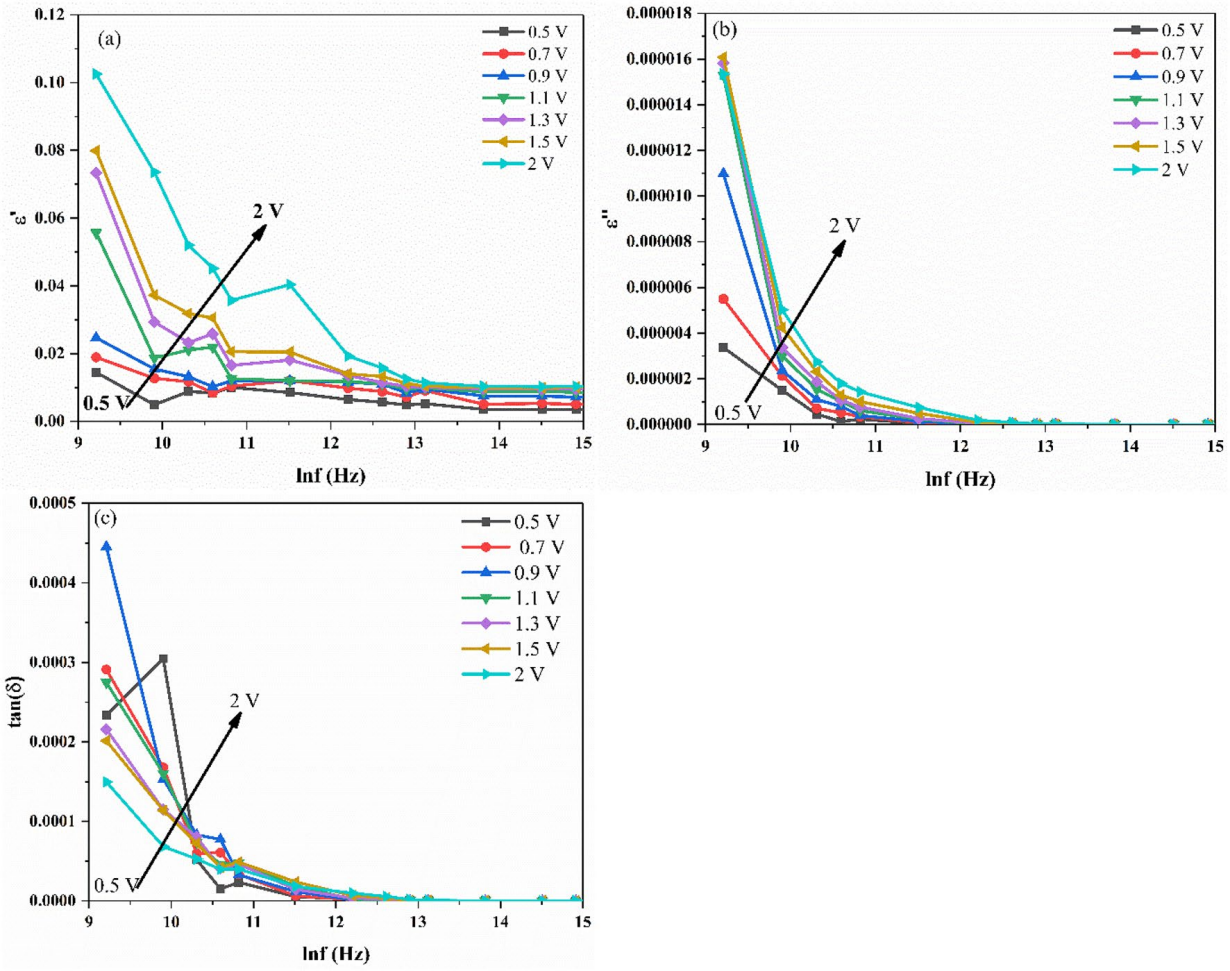
Plot of (**a**) $\upepsilon$′ versus lnf, (**b**) $\upepsilon$″ versus lnf, (**c**) tan(δ) versus lnf for the Ag/TiO_2_-NW/Ge-NW/Si (MOS) device.

Both the real and imaginary parts are calculated from the measured C and G/ω. The dielectric loss is expressed as the energy loss caused by the heating of a dielectric material in a variable electric field^26^. The loss factor is known as the energy spent at the dielectric to avoid bound charge displacement to be in phase with the field alternations^26^. From Fig. 6a-c, it is observed that the $\upepsilon$′, $\upepsilon$″ and tan(δ) values show independent behaviour from frequency in the inversion region and then starts to increase from the depletion region to the accumulation region. Furthermore, the $\upepsilon$′, $\upepsilon$″ and tan(δ) values decrease with increasing frequency. This behaviour can be explained by the fact that when the frequency is increased, the interfacial dipoles in the dielectric have less time to orient themselves in the direction of the alternating electric field and alternatively, the polarization decreases with the increase in frequency and remains constant^26^. This implies that at higher frequencies, the contribution of D_it_ and dipole polarization could be neglected. In other words, the decrement in the $\upepsilon$′ is due to the fact that the dipoles do not have enough time to orient themselves in the direction of electric field and the D_it_ cannot follow the ac signal. Furthermore, the mix-phase established from the XRD plot, where both anatase and rutile type TiO_2_ are reported might also be the reason behind the decrement in the $\upepsilon$′^33^. The broad peak observed in the tan(δ) plot at frequencies less than 200 kHz could be attributed to the relaxation process and the D_it_. In addition, the behaviour of decrease in the values of the measured capacitance (C), dielectric constant ($\upepsilon$′) and dielectric loss ($\upepsilon$″) decreases with the increase in frequency is attributed to the presence of the interfacial polarization mechanism^28^. Moreover, to show the effect of voltage on the dielectric parameters, frequency dependent plots are shown in Fig. 7a-c at different bias voltage, respectively. It is observed that the decrease in values of $\upepsilon$′, $\upepsilon$″ and tan(δ) with the increase in frequency is due to the decrease in polarization with the increase in frequency and the values remains constant at high frequency. This implies that the contribution of dipole polarization and D_it_ could be neglected at high frequency. Therefore, the D_it_ cannot follow the ac signal and the absence of any interfacial polarization mechanism in the MOS device makes the contribution to C, $\upepsilon$′ and $\upepsilon$″ negligible at high frequencies (> 200 kHz). The low frequency dielectric behaviour of the MOS device can be attributed to four possible mechanisms: electrode interface, dc conductivity, dipole-orientation and charge carriers^34^.

## Experimental procedure

TiO_2_-NW/Ge-NW is fabricated on a 1 cm × 1 cm n-type Si (100) substrate using the GLAD technique incorporated into an electron-beam evaporator (Vacuum Coating Unit Model-BC-300). Using an ultra-sonicator, the Si substrates are cleaned in a 3-step sequence using electronic grade acetone, methanol, and rinsed with de-ionized (DI) water. During the synthesis process, the base pressure of ~ 2 × 10^–6^ mbar is maintained inside the electron-beam chamber. A deposition rate of 0.5 Å s^−1^ is maintained during the synthesis of TiO_2_-NW/Ge-NW using a digital thickness monitor (DTM). Firstly, a thin-film (TF) layer of Ge (30 nm) is deposited over the Si substrate using a pure 99.999% Ge source. Next the substrate is azimuthally rotated at.

**Figure 8:**
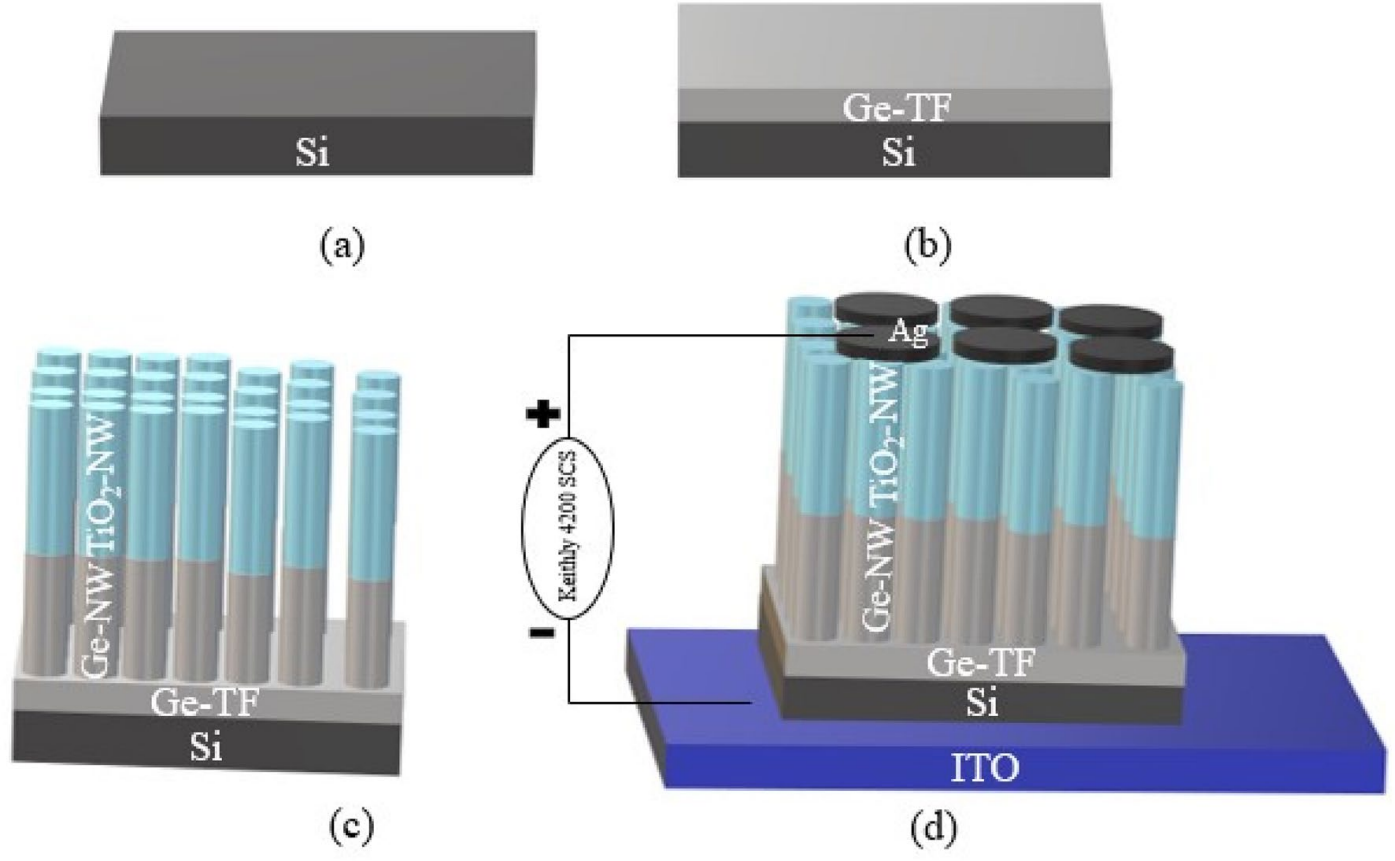
(**a**) Si wafer, (**b**) Synthesis of Ge-TF, (**c**) Synthesis of TiO_2_-NW/Ge-NW/Si using GLAD technique, (**d**) Synthesis of Ag/TiO_2_-NW/Ge-NW/Si (MOS) device schematic.

30 rpm with substrate holder kept inclined at 85° with respect to the source where Ge-NW (200 nm) was synthesized. Subsequently the TiO_2_-NW (200 nm) is fabricated using pure 99.999% TiO_2_ source over the Ge-NW to obtain coaxial TiO_2_-NW/Ge-NW assembly. Finally, silver (Ag) metal contacts are fabricated using an aluminum mask with a hole area ~ 7 mm^2^ on both the samples. The schematic of the Ag/TiO_2_-NW/Ge-NW/Si MOS device is given in Fig. 8a-d. The electrical characterization of the devices is performed using a Keithly 4200 SCS from 10 kHz to 5 MHz.

## Conclusion

The electrical and dielectric parameters of a GLAD synthesized Ag/TiO_2_-NW/Ge-NW/Si (MOS) device have been studied over a wide frequency and voltage ranges. It has been determined from the measured C and G/ω behaviour of the MOS device that the parameters were dependent on the applied frequencies and voltages. In addition, the wide dispersion exhibited in both C and G/ω curves in the depletion region was mainly attributed to the existence of the D_it_. Furthermore, the measured C and G/ω behaviour with the applied frequency and voltage were dependent on the D_it_, R_s_ and the polarization process. The D_it_ and R_s_ parameters were computed using the Hill-Coleman and Nicollian–Brews methods in wide frequency and voltage ranges. The dielectric properties analysis of the MOS device established that the $\upepsilon$′, $\upepsilon$″ and tan(δ) parameters were dependent on frequency and that the values decrease with the increase in frequency. Furthermore, it is observed at higher frequencies (> 200 kHz), the $\upepsilon$′ and $\upepsilon$″ values remains constant which is attributed to the interfacial polarization. Moreover, the decrement in the $\upepsilon$′ value for the MOS device is also due to the mix-phase of anatase and rutile type TiO_2_ present in the structure as determined from the XRD study reported earlier. Therefore, the study of the proposed MOS device has highlighted that it offers improved device capability for opto-electronics applications and also the potential for further improvement to obtain better device performance.

## Data Availability

The datasets generated during and/or analysed during the current study are available from the corresponding author on reasonable request.
